# Effectiveness of Photobiomodulation and Exercise‐Based Rehabilitation on Pain and Functional Recovery in Patients With Rotator Cuff Pathology

**DOI:** 10.1111/wrr.70043

**Published:** 2025-05-15

**Authors:** G. Arun Maiya, Anupama Harihar, Grace Maria Joseph, Esha Arora, Praveen Arany, Rene Jean Bensadoun, Nicolette Nadene Houreld, Liisa Laakso

**Affiliations:** ^1^ Centre for Podiatry & Diabetic Foot Care and Research, Department of Physiotherapy, Manipal College of Health Profession Manipal Academy of Higher Education Manipal India; ^2^ Department of Physiotherapy Asian Institute of Medical Science and Technology Bedong Kedah Malaysia; ^3^ Department of Oral Biology, Surgery and Biomedical Engineering University at Buffalo Buffalo New York USA; ^4^ Department of Oncology Radiology Centre De Haute Energie Nice France; ^5^ Department of Health Sciences, Laser Research Centre University of Johannesburg Johannesburg South Africa; ^6^ Menzies Health Institute Queensland Griffith University Gold Coast Australia; ^7^ Mater Research South Brisbane Australia

**Keywords:** exercise rehabilitation, laser, photobiomodulation, rotator cuff pathology

## Abstract

Rotator cuff (RC) pathology encompasses a wide range of conditions, which include bursitis, tendinitis, tendinosis, partial thickness tears and full‐thickness tears. To treat painful musculoskeletal problems, low‐level laser therapy (LLLT) has been employed as a non‐pharmacological alternative. Photobiomodulation (PBM), which uses light‐emitting diodes (LEDs) and other photo‐emitting devices, is a minimally invasive approach used to treat a wide range of conditions. The purpose of this pre‐post study design is to evaluate the effectiveness of PBM and exercise‐based rehabilitation on pain and functional recovery in patients with RC pathology. Twenty of the thirty‐seven patients who were tested for shoulder disorders and found to have RC pathology were included in the study. The patients' pain levels were measured using the Numerical Pain Rating Scale (NPRS) both at baseline and 6 weeks later. The mean ± standard deviation of NPRS was calculated, data was checked for normal distribution, and the Wilcoxon rank test was conducted to compare the values. Our study showed a statistically significant reduction in pain scores from baseline (7.33 ± 0.79) to 6 weeks (2.50 ± 0.69), *p* < 0.001 of PBM and exercise‐based rehabilitation. The knowledge about the evidence regarding the effectiveness of PBM, along with exercise‐based rehabilitation, is critical.

## 1 Introduction

Shoulder pain and dysfunction are among the most common musculoskeletal health problems, affecting 16%–21% of adults and growing more common as people get older. Rotator cuff (RC) tears are among the most common shoulder problems [1]. RC disorders encompass a wide range of pathological conditions, which include bursitis, tendinitis, tendinosis, partial thickness tears and full‐thickness tears [2]. In asymptomatic people with a mean age of 44.3 years, magnetic resonance imaging scans showed the prevalence of full‐thickness tears was 10.3%, and in symptomatic patients, 40.8% [3]. Interestingly, poor posture has also been shown to be a predictor of RC disease. Tears were present in 65.8% of patients with kyphotic‐lordotic postures, 54.3% with flat‐back postures and 48.9% with sway‐back postures; tears are present in only 2.9% of patients with ideal alignment [4]. An individual suffering from a RC injury may experience pain, tenderness, difficulty lifting their hand and an inability to carry out daily tasks due to the specific injury to their shoulder [5]. Young individuals who participate in sports are also more likely to sustain a RC injury. This injury affects quality of life and is quite common in older people. It happens during an upper extremity‐related sporting activity and causes pain, weakness, soreness and restriction in shoulder movement [5]. There are two types of causes for this condition: non‐traumatic and traumatic. Non‐traumatic causes include muscle weakness and internal bony changes (osteoarthritis). Traumatic causes include genetic predisposition, extrinsic impingement and biomechanical imbalance from the RC structure, intrinsic degeneration from the muscles and tendon itself, and comorbidities [6].

Low‐level laser therapy (LLLT), also known as photobiomodulation (PBM), has been used as a non‐pharmacological alternative to treat painful musculoskeletal conditions [7]. PBM uses light‐emitting diodes (LEDs) and other photo‐emitting devices and is a minimally invasive approach used to treat a wide range of conditions. With wavelengths ranging from 400 to 1200 nm, the red‐infrared portion of the electromagnetic spectrum contains the most researched phototherapies that aid inpatient rehabilitation [8, 9]. PBM reduces inflammation by improving microcirculation and stimulating angiogenesis while inhibiting inflammatory cells and cytokines and enhancing adenosine triphosphate synthesis [10]. Similarly, it is used to treat other musculoskeletal conditions, such as fibromyalgia patients, to enhance their quality of life and reduce pain. Also, it is used in addition to exercises to treat lumbar disk herniation [11].

Using infrared LEDs for PBM treatments has been shown to modify cellular and molecular metabolism, reducing pro‐inflammatory cytokine levels (mRNA COX‐2 and mRNA) and neutrophil and histiocytic counts in the treated area [12]. Given that there is limited research on PBM treatment for RC injuries and shoulder conditions, particularly in combination with exercise there is a need to evaluate the effectiveness of PBM along with exercise‐based rehabilitation based on the pain and functional activity levels in patients with RC injuries. This study evaluated the effectiveness of PBM and exercise‐based rehabilitation on pain and functional recovery in patients with RC pathology by assessing pain and range of motion (ROM) of affected shoulder joints.

## 2 Materials and Methods

### 2.1 Study Design

Following institutional ethical approval for this study (IEC: 846/2017), pre‐post intervention assessments were performed.

### 2.2 Study Setting and Participants

This study was conducted from March 2023 to September 2023 in a tertiary care hospital. The study was conducted in patients with RC pathology. Patients with shoulder pain and restricted shoulder mobility were screened. Participants were included as per the selection criteria of age limit being 18–70 years, either gender with a numerical pain rating score (NPRS) of five score and above, less than 50% of ROM of shoulder joint, aetiology of injury being idiopathic, traumatic or degenerative, patients with RC pathology coming to the department of physiotherapy. Jobe's test was performed to clinically assess the diagnosis of RC pathology [13], and the diagnosis was confirmed with ultrasound imaging by an orthopaedic surgeon specialising in shoulder pathologies.

### 2.3 Intervention

**FIGURE 1:**
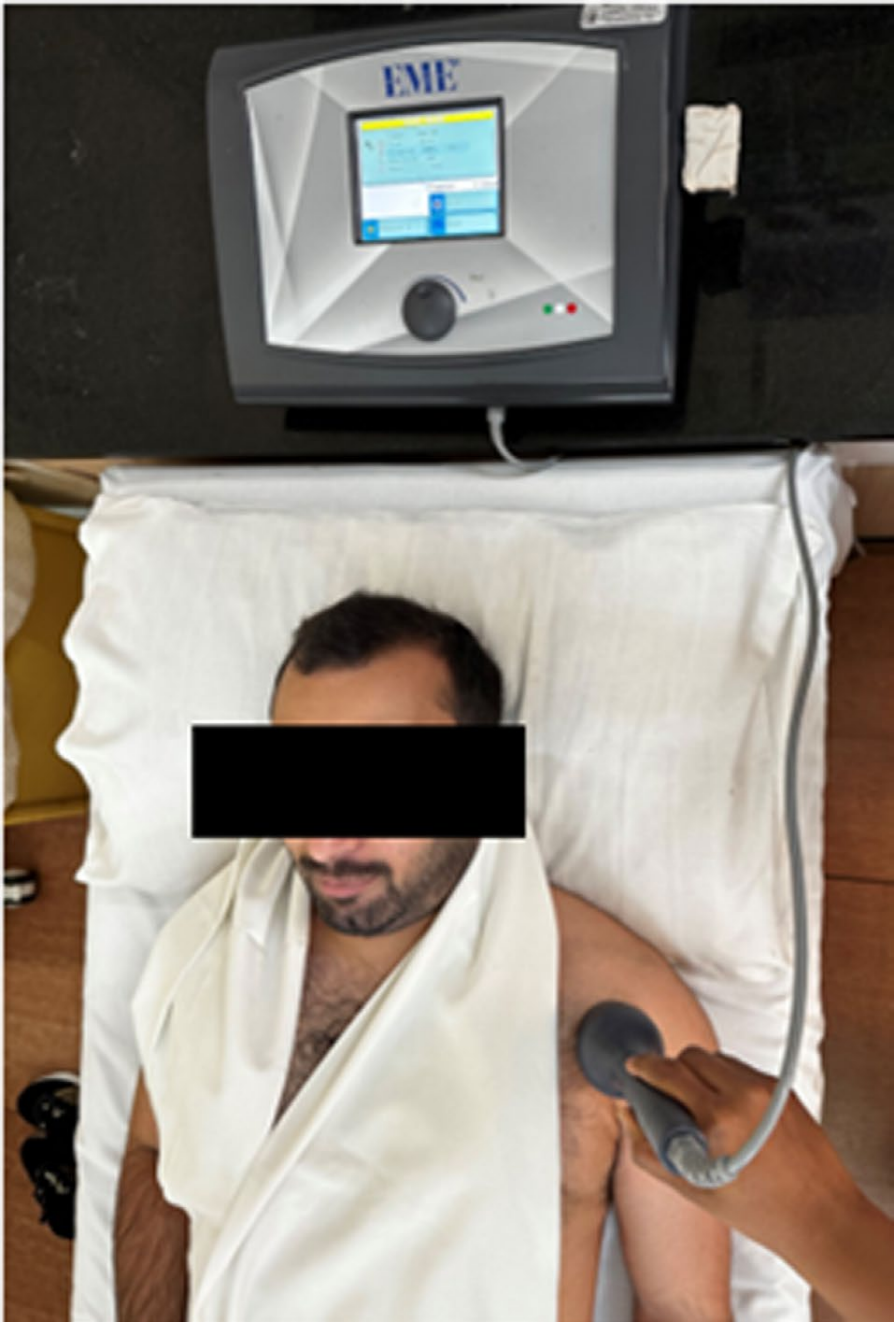
Application of photobiomodulation therapy using probe laser equipment.

After assessing the patient's baseline NPRS and functional limitations, the participants were exposed to irradiation of PBM treatment for the duration of 10 days, after which the patients were started with structured exercise rehabilitation, which was prescribed for 4 weeks. PBM treatments were given with a cluster probe named Lasermed 2200‐LT1372 manufactured by EME ITALY. While irradiation, the participants were instructed to expose the affected shoulder joint, and protective goggles were advised to be worn throughout the PBM session. The irradiation was given using the probe to the anterior joint line region and lateral aspect of the affected shoulder joint using the following parameters: A total of 19 diode LED cluster probes of 660 nm. The probe is equipped with a 10 × 660 nm LED that has an average power of 10 mW and an active area of 0.2 cm^2^. The power density (irradiance) of the LEDs is 50 mW/cm^2^. The probe laser was applied for 4 min at 156 Hz for each point as shown in Figure 1.

The exercise‐based rehabilitation included, initially, submaximal isometric exercises for the muscles‐deltoid, biceps, triceps and scapular stabilisers, followed by restoring full passive and active ROM exercises for the shoulder joint and progressive gentle strengthening of RC muscles [14].

### 2.4 Outcome Assessments

All measurements were taken at baseline and after 6 weeks of the intervention including PBM and structured exercise‐based rehabilitation. The pain was measured using a patient‐reported NPRS, and functional movements were assessed by evaluating the range of all shoulder movements using a standardised universal goniometer.

### 2.5 Statistical Analysis

Data were analysed using Jamovi 2.4.11 software. Descriptive statistics were done for the demographic characteristics. Shapiro– Wilk test was used to test normality. Wilcoxon signed rank test was used to compare the means of pre‐ and post‐intervention. Data was considered statistically significant when *p* < 0.05.

## 3 Results

### 3.1 Demographic Data

A total of 37 subjects with shoulder were screened, out of which 20 were included in the study, who were diagnosed with RC injuries, and the patient's diagnoses were confirmed by an orthopaedic surgeon after performing the physical test along with ultrasound imaging. Seventeen participants were excluded as they did not meet the inclusion criteria.

The included participants were diagnosed with RC partial or full tear injury (*n* = 6), shoulder impingement syndrome (*n* = 4), bursitis (*n* = 4), tendinitis (*n* = 3) or tendinopathy (*n* = 3). However, the following were the reasons for the exclusion of 17 participants: the ROM was more than 50% of normal (*n* = 8), pain score on the NPRS of less than 5 unit (*n* = 5), due to associated neck pain or upper back pain (*n* = 3); and also, exceeding the age limit of 60 years (*n* = 1) (Table 1).

**TABLE 1:** Demographic characteristics of patients with rotator cuff pathology.

Characteristics	Mean ± SD of participants (*n* = 20)
Age (years)	47.3 ± 9.5
Gender	Male (*n* = 15)
	Female (*n* = 5)
Duration of pain (in months)	6.4 ± 3.3
Aetiology of pain (*n*)	
Idiopathic	6 (30%)
2 Traumatic	9 (45%)
3 Degenerative	5 (25%)
Comorbidities (*n*)	
Type 2 Diabetes Mellitus	6 (30%)
2 Hypertension	5 (25%)
3 Hypothyroidism	2 (10%)
4 Hyperlipidaemia	3 (15%)
5 No comorbidities	4 (20%)

### 3.2 Pain Assessments

The pain severity based on the NPRS scale is categorised as 0 = *no pain*, 1–3 = *mild*, 4–6 = *moderate* and 7–10 = *severe* [15]. In the present study, 80.5% of patients reported severe pain prior to intervention. Following the treatment, approximately 88.8% of the population experienced a reduction in pain intensity to 2 out of 10 score on NPRS. The mean ± standard deviation of the subjects' pain level that is, pre‐intervention NPRS values being 7.33 ± 0.79 and post‐intervention NPRS values was 2.50 ± 0.69 following 6 weeks of PBM and exercise based rehabilitation. There was a statistically significant difference of *p* < 0.001 on comparison of the pre‐post intervention values of NPRS.

### 3.3 Functional Assessments

There was a statistically significant improvement of < 0.001 in the abduction, internal rotation and external rotation of ROM of the shoulder joint after the treatment of PBM and structured exercise protocol. Although flexion and extension ROM of the shoulder did not show a significant difference, a minimal increased range was noted (Table 2).

**TABLE 2:** Range of motion pre and post‐PBM and exercise‐based rehabilitation.

Movement	Pre (mean ± SD)	Post (mean ± SD)	*p*
Flexion	161° ± 13.3°	161° ± 18.1°	0.78
Extension	44° ± 9.8°	47° ± 12.8°	0.36
Abduction	120° ± 13.1°	170° ± 12.1°	< 0.001
Internal rotation	54.6° ± 9.6°	81.3° ± 6.4°	< 0.001
External rotation	54.4° ± 10.3°	81.3° ± 6.4°	< 0.001

## 4 Discussion

The spectrum of injuries to the RC includes partial tears, tendinopathy, injury, and ultimately, full tears. We conducted a clinical study to assess the benefits of photobiomodulation and exercise‐based therapy for RC pathologies. The study results show that the intervention resulted in a significant reduction in the NPRS level of patient pain intensity. The difference in pain outcome reported was five units on the NPRS from baseline and post‐intervention. This indicates a 50% reduction in pain post‐intervention using PBM therapy and exercises. Previous literature suggests that non‐surgical physical therapy treatment of 6–12 weeks has been an effective treatment for full‐thickness RC tears in 75% of patients up to 2 years of follow‐up [3]. The surgical intervention will pose long‐term structural and clinical success rates, present potential risks, lead to a longer duration of recovery, and require post‐operative rehabilitation for satisfactory improvement [16].

A study by Scaldaferri Martins et al. showed the improvement in ROM, pain, strength and quality of life in individuals with RC retinopathy post‐PBM intervention, along with therapeutic ultrasound in 4 weeks [8]. Similarly, a PBM therapy of pulsed 890 nm, when given for 8 min in patients with shoulder pain due to subacromial syndrome, proved more effective in the improvement of pain and ROM than exercise therapy alone [17]. Another study by Eslamian et al. [18] found that incorporating PBM into a combination treatment of exercises, along with heat therapy, ultrasound and TENS, resulted in greater pain relief than the combination therapy alone in a group of 50 patients with RC tendinitis. Overall, multiple evidences determine the effect of PBM in reducing pain, improving ROM of the shoulder, and quality of life. Contradictorily, a few studies showed no change in ROM after PBM therapy, although pain was improved after a month's follow‐up [19, 20].

A systematic review by Marco Castaldo et al. in 2023, also showed that the use of LLLT, in combination with therapeutic exercises, was effective in treating shoulder pathology. The review found that LLLT combined with therapeutic exercise resulted in a significant decrease in pain severity and improved shoulder function [21]. Similarly, Nicholas Tripodi et al. conducted a systematic review in 2021 that demonstrated the benefits of LLLT and red‐infrared photobiomodulation on pain and function in tendinopathy. The review found that PBM is increasingly being used to treat musculoskeletal disorders and that adjunctive exercise‐adjunctive treatment with PBM resulted in reduced pain intensity and improved function and quality of life in patients with tendinopathies [19]. However, the effectiveness of PBM treatment in terms of biomechanical pathways requires further investigation.

Pain results from a stimulus that triggers the rapid propagation of action potentials along a nerve cell. This propagation is caused by the expulsion of positively charged sodium ions (Na+) and the influx of potassium (K+) ions into the nerve cell. The absorption of PBM by nerve cell receptors within the bilipid cellular membrane potentially holds significant potential for pain management. Lipids absorb best between 905 and 910 nm in wavelengths [12]. Upon absorption, the light generated by PBM therapy initiates a series of physiological effects that increase the permeability of the cellular membrane. This mechanism facilitates the reabsorption of Na+ ions into the cell and the removal of K+ ions, thereby maintaining the equilibrium of the sodium‐potassium pump. This action effectively eliminates the pain signal at its origin, rendering PBM therapy a viable therapeutic option [12]. Exercise training regimens can also be made more effective with PBM. Thus, it should come as no surprise that PBM can aid in the healing of muscle injuries as well as lessen discomfort and soreness in the muscles following strenuous exercise. Numerous studies conducted on animals have examined indicators of oxidative stress and inflammation in muscle tissue taken from animals that have been sacrificed [12].

A recent study conducted by Martins et al. in 2022, analysed pain relief and functional recovery in patients with RC tendinopathy through therapeutic ultrasound and PBM. The study was a randomised controlled trial and compared the effectiveness of different treatments. The results of the study suggest that adding infrared and red LED treatments to ultrasound can improve the quality of life, reduce pain intensity, increase shoulder ROM, and enhance muscle strength for patients suffering from RC tendinopathy. The researchers concluded that infrared LED treatments could be a promising therapy for clinical practice [8, 22].

The major limitation of our study was the smaller sample size and the lack of a control group. Hence, future research can be recommended on a randomised controlled trial in evaluating the effect of PBM alone, which can provide clarity on the independent advantages of PBM in shoulder pathology and various musculoskeletal conditions. In conclusion, the integration of PBM therapy with exercise‐based rehabilitation demonstrated a substantial improvement in pain reduction and improved ROM in the shoulder joint, as indicated by the results, and exercise‐based rehabilitation shows promising results in improved functional activity in clinical practice.

## Author Contributions

**G. Arun Maiya:** conceptualization. **Esha Arora**, **Praveen Arany**, **Rene Jean Bensadoun**, **Nicolette Nadene Houreld** and **Liisa Laakso:** methodology, writing – review and editing. **G. Arun Maiya:** formal analysis and investigation. **Anupama Harihar** and **Grace Maria Joseph:** writing – manuscript draft preparation. **Anupama Harihar** and **Grace Maria Joseph:** exercise‐based rehabilitation. **G. Arun Maiya:** supervision. All authors contributed equally to the present review.

## Conflicts of Interest

The authors declare no conflicts of interest.

## Data Availability

The data is available with the corresponding author and will be shared upon reasonable request.

## References

[1] D. Burns , H. Razmjou , J. Shaw , et al., “Adherence Tracking With Smart Watches for Shoulder Physiotherapy in Rotator Cuff Pathology: Protocol for a Longitudinal Cohort Study,” JMIR Research Protocols 9, no. 7 (2020): e17841, 10.2196/17841. 32623366 PMC7381014

[2] K. A. R. Kemp , D. M. Sheps , C. Luciak‐Corea , F. Styles‐Tripp , J. Buckingham , and L. A. Beaupre , “Systematic Review of Rotator Cuff Tears in Workers'compensation Patients,” Occupational Medicine 61, no. 8 (2011): 556–562, 10.1093/occmed/kqr068. 22016341

[3] J. E. Kuhn , “Prevalence, Natural History, and Nonoperative Treatment of Rotator Cuff Disease,” Operative Techniques in Sports Medicine 31, no. 1 (2023): 150978, 10.1016/j.otsm.2023.150978.

[4] T. May and G. M. Garmel , Rotator Cuff Injury (StatPearls, 2023). 31613444

[5] S. Zafar and S. Kumar , “Therapeutic Effects of Moist Hot Packs With Laser and Ultrasound in Rotator Cuff Injury,” International Journal of New Technology and Research 3 (2017): 263218, https://www.researchgate.net/profile/Suraj‐Kumar‐30/publication/320711258_Therapeutic_Effects_of_Moist_Hot_Packs_with_Laser_and_Ultrasound_in_Rotator_Cuff_Injury/links/59f70e500f7e9b553ebd52f7/Therapeutic‐Effects‐of‐Moist‐Hot‐Packs‐with‐Laser‐and‐Ultrasound‐in‐Rotator‐Cuff‐Injury.pdf.

[6] L. J. Soslowsky , J. E. Carpenter , J. S. Bucchieri , and E. L. Flatow , “Biomechanics of the Rotator Cuff,” Orthopedic Clinics of North America 28 (1997): 17–30. 9024428 10.1016/s0030-5898(05)70261-3

[7] S. Haslerud , L. H. Magnussen , J. Joensen , R. A. B. Lopes‐Martins , and J. M. Bjordal , “The Efficacy of Low‐Level Laser Therapy for Shoulder Tendinopathy: A Systematic Review and Meta‐Analysis of Randomized Controlled Trials,” Physiotherapy Research International 20 (2015): 108–125. 25450903 10.1002/pri.1606

[8] J. P. S. Martins , C. J. de Lima , A. B. Fernandes , L. P. Alves , O. P. Neto , and A. B. Villaverde , “Analysis of Pain Relief and Functional Recovery in Patients With Rotator Cuff Tendinopathy Through Therapeutic Ultrasound and Photobiomodulation Therapy: A Comparative Study,” Lasers in Medical Science 37 (2022): 3155–3167. 35648258 10.1007/s10103-022-03584-2

[9] S. R. Tsai and M. R. Hamblin , “Biological Effects and Medical Applications of Infrared Radiation,” Journal of Photochemistry and Photobiology. B, Biology 170 (2017): 197. 28441605 10.1016/j.jphotobiol.2017.04.014 PMC5505738

[10] A. A. Vanin Vanin , T. De Marchi , S. S. Tomazoni , et al., “Pre‐Exercise Infrared Low‐Level Laser Therapy (810 Nm) in Skeletal Muscle Performance and Postexercise Recovery in Humans, What Is the Optimal Dose? A Randomized, Double‐Blind, Placebo‐Controlled Clinical Trial,” Photomedicine and Laser Surgery 34 (2016): 473–482, https://home.liebertpub.com/pho. 27575834 10.1089/pho.2015.3992

[11] M. Ester , I. De Mendes Carvalho , R. De Mendes Carvalho , et al., “Low Intensity Laser and LED Therapies Associated With Lateral Decubitus Position and Flexion Exercises of the Lower Limbs in Patients With Lumbar Disk Herniation: Clinical Randomized Trial,” Lasers in Medical Science 31 (2016): 1455–1463. 27379776 10.1007/s10103-016-2009-5

[12] M. R. Hamblin , “Mechanisms and Applications of the Anti‐Inflammatory Effects of Photobiomodulation,” AIMS Biophysics 4 (2017): 337–361. 28748217 10.3934/biophy.2017.3.337 PMC5523874

[13] N. B. Jain , J. Luz , L. D. Higgins , et al., “The Diagnostic Accuracy of Special Tests for Rotator Cuff Tear: The ROW Cohort Study,” American Journal of Physical Medicine & Rehabilitation 96, no. 3 (2017): 176–183, 10.1097/PHM.0000000000000566. 27386812 PMC5218987

[14] T. S. Ellenbecker and G. J. Davies , “Current Concepts in Rehabilitation of Rotator Cuff Pathology: Nonsurgical and Postoperative Considerations,” in Orthopaedic Knowledge Update: Sports Medicine 5 (Wolters Kluwer Health, 2018), 311–328.

[15] A. M. Boonstra , R. E. Stewart , A. J. Albère , et al., “Cut‐Offpoints for Mild, Moderate, and Severe Pain on the Numeric Rating Scale for Pain in Patients With Chronic Musculoskeletal Pain: Variability and Influence of Sex and Catastrophizing,” Frontiers in Psychology 7 (2016): 212592. 10.3389/fpsyg.2016.01466 PMC5043012 27746750

[16] C. Schmidt , C. Jarrett , and B. T. Brown , “Management of Rotator Cuff Tears,” Journal of Hand Surgery 40 (2015): 399–408, https://www.sciencedirect.com/science/article/pii/S0363502314008934?casa_token=UKizsszTEc8AAAAA:Yjq3yixlFI1sagpJWAyixCRjm5Hp_z13i3BuptAUXT2oyepYFWLEf2FGWSthCrAHVR6uDDg4hD8. 25557775 10.1016/j.jhsa.2014.06.122

[17] S. M. J. Abrisham , M. Kermani‐Alghoraishi , R. Ghahramani , L. Jabbari , H. Jomeh , and M. Zare , “Additive Effects of Low‐Level Laser Therapy With Exercise on Subacromial Syndrome: A Randomised, Double‐Blind, Controlled Trial,” Clinical Rheumatology 30 (2011): 1341–1346. 21538218 10.1007/s10067-011-1757-7

[18] F. Eslamian , S. Kazem Shakouri , M. Ghojazadeh , et al., “Effects of Low‐Level Laser Therapy in Combination With Physiotherapy in the Management of Rotator Cuff Tendinitis,” Lasers in Medical Science 27 (2012): 951–958. 22052627 10.1007/s10103-011-1001-3

[19] N. Tripodi , J. Feehan , M. Husaric , F. Sidiroglou , and V. Apostolopoulos , “The Effect of Low‐Level Red and Near‐Infrared Photobiomodulation on Pain and Function in Tendinopathy: A Systematic Review and Meta‐Analysis of Randomized Control Trials,” BMC Sports Science, Medicine and Rehabilitation 13, no. 1 (2021): 91, 10.1186/s13102-021-00306-z. PMC8364035 34391447

[20] C. L. R. Leotty , M. M. C. Lima , and F. X. de Araújo , “Effect of Low‐Level Laser Therapy on Pain and Function of Patients With Shoulder Tendinopathy: A Systematic Review,” Physiotherapy and Research 27 (2020): 210–217, 10.1590/1809-2950/19027827022020.

[21] M. Castaldo , D'O. A. De Angelis , P. Gnessi , et al., “A Systematic Review on Low‐Level Laser Therapy in the Management of Shoulder Impingement Syndrome,” Applied Sciences 13 (2023): 3536.

[22] A. AA‐Shenqiti and J. Oldham , “The Use of Low‐Level Laser Therapy (LLLT) in the Treatment of Trigger Points That Are Associated With Rotator Cuff Tendonitis,” *Proceedings*, (2003), 5287, 91–101, 10.1117/12.544884.

